# The immunoreactivity of GLI1 and VEGFA is a potential prognostic factor in kidney renal clear cell carcinoma

**DOI:** 10.1186/s12885-023-11622-7

**Published:** 2023-11-14

**Authors:** Anna Kotulak-Chrzaszcz, Jacek Kiezun, Mateusz Czajkowski, Marcin Matuszewski, Jakub Klacz, Bartlomiej E. Krazinski, Janusz Godlewski, Zbigniew Kmiec, Piotr M. Wierzbicki

**Affiliations:** 1 Department of Histology, Faculty of Medicine, Medical University of Gdansk, 1 Debinki Street, Gdansk, 80211 Poland; 2 Department of Human Histology and Embryology, School of Medicine, Collegium Medicum, University of Warmia and Mazury in Olsztyn, Olsztyn, 10082 Poland; 3 Department of Urology, Faculty of Medicine, Medical University of Gdansk, Gdansk, 80402 Poland

**Keywords:** KIRC, Sonic hedgehog pathway, SHH, GLI1, VEGFA, Prognostic factors, IHC

## Abstract

Kidney renal clear cell carcinoma (KIRC) is the most common type of kidney cancer and its pathogenesis is strongly associated with VHL–HIF–VEGF signaling. SHH ligand is the upstream SHH pathway regulator, while GLI1 is its major effector that stimulates as a transcription factor, i.a. expression of *VEGFA* gene. The aim of present study was to assess the prognostic significance of SHH, GLI1 and VEGFA immunoreactivity in KIRC tissues. The analysis included paired tumor and normal samples from 34 patients with KIRC. The immunoreactivity of SHH, GLI1 and VEGFA proteins was determined by immunohistochemical (IHC) renal tissues staining. The IHC staining results were assessed using the immunoreactive score (IRS) method which takes into account the number of cells showing a positive reaction and the intensity of the reaction. Increased GLI1 protein immunoreactivity was observed in KIRC tissues, especially in early-stage tumors, according to the TNM classification. Elevated expression of the VEGFA protein was noted primarily in high-grade KIRC samples according to the Fuhrman/WHO/ISUP scale. Moreover, a directly proportional correlation was observed between SHH and VEGFA immunoreactivity in TNM 3 + 4 and Fuhrman/ISUP/WHO 3 + 4 tumor tissues as well as in samples of patients with shorter survival. We also observed an association between shorter patient survival as well as increased and decreased immunoreactivity, of the VEGFA and GLI1, respectively. The aforementioned findings suggest that the expression pattern of SHH, GLI1 and VEGFA demonstrates prognostic potential in KIRC.

## Introduction

According to the GLOBOCAN statistics estimated number of renal cell carcinoma (RCC) new cases in 2020 amounted to 431,288 [1]. Kidney renal clear cell carcinoma (KIRC) is the most common type (70–80%) of renal cancer [2] and its development and progression is strongly associated with the von Hippel–Lindau - hypoxia inducible factor - vascular endothelial growth factor (VHL–HIF–VEGF) pathway [3]. The identification of new molecular targets for kidney cancer growth could benefit the therapeutic process of this cancer [4].

One of the potential tumor-growth stimulating mechanism is the Sonic Hedgehog (SHH) signaling with its major components: up-stream signaling regulator SHH ligand, and the main effector, which is the Glioma-associated oncogene family zinc finger 1 (GLI1) transcription factor. Although physiologically, the pathway is responsible for regulation of embryonic morphogenesis [5, 6], its abnormal activation has been implicated in various types of cancers, i.a. medulloblastoma [7], basal cell carcinoma [8, 9], breast [10–12] and colorectal cancers [13–15]. The activation of the SHH pathway depends on the SHH glycoprotein concentration gradient in the extracellular matrix [16, 17].

The SHH glycoprotein is a molecule that plays an important role in the embryonic development [17–19]. In adult life, SHH ligand interacts with Patched 1 (PTCH1), a 12-pass transmembrane receptor, and in this way it modifies the conformation of the Smoothened (SMO), a member of the G-protein coupled receptor family [20, 21]. This leads to disintegration of subplasmalemmal Glioma-associated oncogene family zinc finger 2/3- Suppressor of Fused- kinesin family member 7 (GLI2/GLI3-SUFU-KIF7) complex, and GLI2 as well as GLI3 undergo proteolysis as well as phosphorylation during the translocation to the cell nucleus [17, 22]. Activated GLI2 or GLI3 act as zinc finger transcription factors (TFs) for several target genes, i.a. *GLI1* and *VEGFA*. Both GLI2 and GLI3 contain transcription activating and inhibitory domains, while GLI1 TF possesses the activating sequence only [23]. Enhanced expression of the *GLI1* gene results in a positive feedback loop for the SHH signaling pathway [24]. The activity of the SHH signaling is strongly associated with primary cilia, nonmotile projections that are noted to be present on wide range of mammalian cells. Upon binding of SHH to PTCH1, the repression of SMO by PTCH1 is relieved, allowing SMO to enter the cilium and activates GLI TFs [25].

*VHL* is a tumor suppressor gene associated with Von Hippel–Lindau disease, but also it is widely mutated in sporadic KIRC [26, 27]. In normoxia Von Hippel–Lindau (VHL) protein is a part of ligase ubiquitin complex that stimulates proteosomal degradation of Hypoxia inducible factor (HIF) 1-alpha and HIF2-alpha [28]. However, in hypoxic or *VHL*-mutated conditions, HIFs acts as TFs for several target genes, encoding pro-angiogenic factors [29]. One of the most significant is Vascular Endothelial Growth Factor A (VEGFA), a member of the platelet-derived growth factor (PDGF)/vascular endothelial growth factor (VEGF) family [30]. Through binding with VEGFR1 and -R2 receptors, VEGFA stimulates proliferation, migration and survival of vascular endothelial cells [31]. Although in the vast majority of KIRC samples an increased amount of VEGFA can be found [3, 32], it is still uncertain whether the VEGFA expression pattern is related to the stage or grade of this cancer type.

VHL-HIF signaling cascade stimulates transcription of *VEGFA* gene under hypoxia or in KIRC, due to inactivation of the *VHL* gene [33]. However, it has been reported that *VEGFA* gene is one of the SHH pathway target gene [24, 34] Our previous results revealed that *SHH* and *GLI1* genes were upregulated especially in early KIRC at the mRNA levels, as well as elevated *VEGFA* mRNA ratio was associated with shorter overall survival (OS) of the patients [3, 35]. Therefore, we decided to assess the immunoreactivity of SHH, GLI1 and VEGFA proteins and analyze the results in terms of their prognostic potential in KIRC.

## Materials and methods

### Patients and samples

KIRC tumor tissues and morphologically unchanged kidney samples were obtained from 34 patients who underwent radical nephrectomy at the Department of Urology, Medical University of Gdańsk (Gdańsk, Poland). The samples were collected over a 4‑year period from 2017 to 2020. The group of 34 patients with KIRC, encompassed 8 women and 26 men (Table 1). The exclusion criteria included: other than KIRC histological subtypes of RCC, multifocal and/or bilateral kidney tumors and Von Hippel‑Lindau disease. The study was approved by the Independent Bioethics Committee for Scientific Research at Medical University of Gdańsk (decision nos. NKEBN/4/2011 and NKBBN/370/2016). Written informed consent was obtained from each patient before surgery.

**Table 1:** Association between SHH, GLI1 and VEGFA protein immunoexpression and clinical data of kidney renal cancer patients

Patients/proteins	Subgroups	SHH IRS value			GLI1 IRS value			VEGFA value		
n = 34		↓	↑	*p*-value	↓	↑	*p*-value	↓	↑	*p*-value
Age (years) Mean ± SD 67.03 ± 9.5 Range: 46–86	≤ 67	5	11	0.703	3	13	0.693	6	10	1.000
	n = 16 (47.06%)	14.71%	32.35%		8.82%	38.24%		17.65%	29.41%	
	> 67	4	14		5	13		7	11	
	n = 18 (52.94%)	11.76%	41.18%		14.71%	38.24%		20.59%	32.35%	
Sex	Female	2	6	1.000	0	8	0.152	3	5	1.000
	n = 8 (23.53%)	5.88%	17.65%		0.00%	23.53%		8.82%	14.71%	
	Male	7	19		8	18		10	16	
	n = 26 (76.47%)	20.59%	55.88%		23.53%	52.94%		29.41%	47.06%	
Tumor size (cm)	≤ 7 cm	6	14	0.704	2	**18**	**0.042**	8	12	1.000
	n = 20 (58.82%)	17.65%	41.18%		5.88%	52.94%		23.53%	35.29%	
	> 7 cm	3	11		6	8		5	9	
	n = 14 (41.18%)	8.82%	32.35%		17.65%	23.53%		14.71%	26.47%	
ISUP Histological grade	1 + 2	3	8	1.000	2	9	1.000	7	4	0.060
	n = 11 (32.35%)	8.82%	23.53%		5.88%	26.47%		20.59%	11.76%	
	3 + 4	6	17		6	17		6	17	
	n = 23 (67.65%)	17.65%	50.00%		17.65%	50.00%		17.65%	50.00%	
TNM stage	non-metastatic	4	7	0.425	1	10	0.228	5	6	0.709
	n = 11 (32.35%)	11.76%	20.59%		2.94%	29.41%		14.71%	17.65%	
	metastatic	5	18		7	16		8	15	
	n = 23 (67.65%)	14.71%	52.94%		20.59%	47.06%		23.53%	44.12%	

High (↑) and low (↓) immunoreactivity groups were classified according to the median IRS values in morphologically unchanged paired kidney tissue. Cut off values of IRS: SHH − 8.08, GLI1–5.00, VEGFA − 3.50. Percentage values of subgroups refer to the total number of patients (n = 34). P‑values were calculated by Fisher’s 2 × 2 test. IRS, immunoreactivity score (range 0–12) was determined as described in Methods. SHH ‑ sonic hedgehog; GLI1 - glioma‑associated zinc finger protein 1; VEGFA - vascular endothelial growth factor A

Small (ca. 5 × 5 × 5 mm) pieces of KIRC tumor tissues and control, morphologically unchanged kidney samples from the same patient, were placed into test tubes in the operating theater, no longer than 20 min after kidney resection. Tissue samples for histopathological assessment and immunohistochemical (IHC) staining were placed in test tubes filled with 5 volumes of 4% buffered formalin (POCh, Poland). The samples were included in the analysis if > 60% cells in the respective histological sections in tumor samples presented characteristic features of KIRC, while all cells of unchanged (control) samples presented normal morphology [36, 37]. If both conditions were not fulfilled, the material was excluded from the study. Tumor stage was assessed according to the Union for International Cancer Control TNM 8th staging edition of RCC guidelines [4]. The degree of tumor malignancy was determined using the Fuhrman or WHO/ISUP grading system [38]. The tissues fixed in 4% buffered formalin were stored at 4 ˚C until further analysis. Tissue samples for IHC were prepared by routine technique that included dehydration, paraffin embedding and cutting into 5 𝜇m-thick sections.

### Immunohistochemistry

IHC staining was performed in Department of Human Histology and Embryology, Faculty of Medicine, University of Warmia and Mazury in Olsztyn, Poland. Immunohistochemical analysis was performed as described previously by Kieżun et al. (2022) with modifications [39]. The sections were subjected to an antigen retrieval procedure by microwaving for 7 min in Retrieval Solution Buffer, pH 6.0 or pH 9.0 (Leica Microsystems, Germany, pH 6.0 for antibodies against GLI1, pH 9.0 for antibodies against SHH and VEGFA), and then incubating with 3% H_2_O_2_ in methanol for 10 min for blocked endogenous peroxidase activity. Next, the unspecific binding sites were blocked with 2.5% normal horse serum (Vector Laboratories, USA) for 30 min. The sections were incubated overnight at 4 °C with rabbit monoclonal anti-human antibodies (all from Abcam, UK, diluted in phosphate-buffered saline, PBS) against GLI1 (1:400, Cat. No. ab289368), SHH (1:1000, Cat. No. 53,281) and VEGFA (1:200, Cat. No. ab52917). Sections were incubated with secondary antibodies (ImmPRESS Universal reagent Anti-Mouse/Rabbit Ig, Vector Laboratories, USA) for 30 min. The specificity of immunohistochemical staining was checked by omitting the primary antibody and by replacing it with the rabbit serum. The sections were visualized with Liquid DAB + Substrate Chromogen System (Dako, USA), then counterstained with hematoxylin (Sigma-Aldrich, USA), dehydrated in ethanol series, rinsed in xylene and mounted in DPX (Sigma-Aldrich). The labelled tissues were photographed using a XC-50 camera (Olympus Corp., Japan) mounted on a direct light BX-41microscope (Olympus Corp.). Concomitantly to IHC, the H&E staining was performed to assess tissue morphology.

### Evaluation of immunohistochemical reactivity

Immunoreactivity of SHH, GLI1 and VEGFA in KIRC tumors and corresponding normal kidney tissue was evaluated by two independent histologists, who were blinded to the patients’ clinical data. In the cases of different assessments, the third histologist checked the sections. The immunoreactive score (IRS) method [40] was used to assess the area of the cells with cytoplasmic positive reaction as well as the color intensity of the reaction. The assessment included three randomly selected parts of the slide, at the magnification of 200×. The IRS scale is based on the percentage of area containing cells with positive reaction, 1 point: 1–10% cells, 2-points: 11–50%, 3 points: 51–80%, and 4 points: over 80% cells with positive reaction, as well as reaction intensity (0, no reaction, 1, low-intensity reaction, 2, moderate-intensity reaction, and 3, intense reaction). The final score depended on both parameters, multiplied percentage of positive cells and intensity of the reaction, and ranged from 0 to 12 points.

### Statistical analysis

Mean IRS value of each KIRC tumor and morphologically unchanged kidney slides underwent statistical analysis including patients’ clinicopathological features. Statistical tests was performed using GraphPad Prism ver. 6.07 (GraphPad Software, Inc., USA) and Statistica ver. 13.3 (StatSoft Ltd., USA) software. The following non‑parametric tests were applied: Wilcoxon signed‑rank if samples were paired, Kruskal-Wallis test for multiple comparison and Spearman’s correlation. The median IRS values for a particular protein in the control group were used as a threshold for the determination of upregulation and downregulation of a given protein immunoreactivity in cancer tissues. In this way, 2 × 2 Fisher’s exact test was performed. Clinical data concerning patients overall survival were analyzed using GraphPad Prism ver. 6.07. For outcome analysis of patients, Kaplan‑Meier survival tests with log‑rank (Mantel‑Cox) tests were performed by GraphPad Prism ver. 6.07. Mantel‑Cox proportional hazard regression model with univariable (first step) and multivariable (second step) tests were applied. Survival associations were presented as hazard ratios (HRs) with their 95% confidence interval (CI) and P‑values using Mantel‑Cox and Kaplan‑Meier estimations [3, 41].

## Results

### Clinicopathological characteristics of the patients

The clinicopathological features of the patients are presented in Table I. The study encompassed 34 patients with KIRC, including 8 woman and 26 men (mean age ± SD, 67.03 ± 9.5 years; median age was 69 years with the range 46–86 years). Sample staging revealed 10 patients as stage I (T1N0M0), 1 as stage II (T2N0M0), 22 as stage III (T1‑2N1M0 or T3N0‑2M0), and 1 as stage IV (T4N0‑2M0 or T1‑4N0‑2M1; according to the Union for International Cancer Control TNM 8th staging edition of renal cell carcinoma guidelines [4]). Local or distant metastases were diagnosed in 23 (68%) patients, at the time of nephrectomy. Histological Fuhrman/WHO/ISUP grading assessment [38] indicated 2 KIRC samples in grade 1, 9 samples in grade 2, 14 samples in grade 3, and 9 samples in grade 4. The mean follow‑up period was 36 months (range, 6‑120 months). The median OS rate was 30 months. All deaths were associated with KIRC progression.

### GLI1 and VEGFA are overexpressed in KIRC

**Fig. 1:**
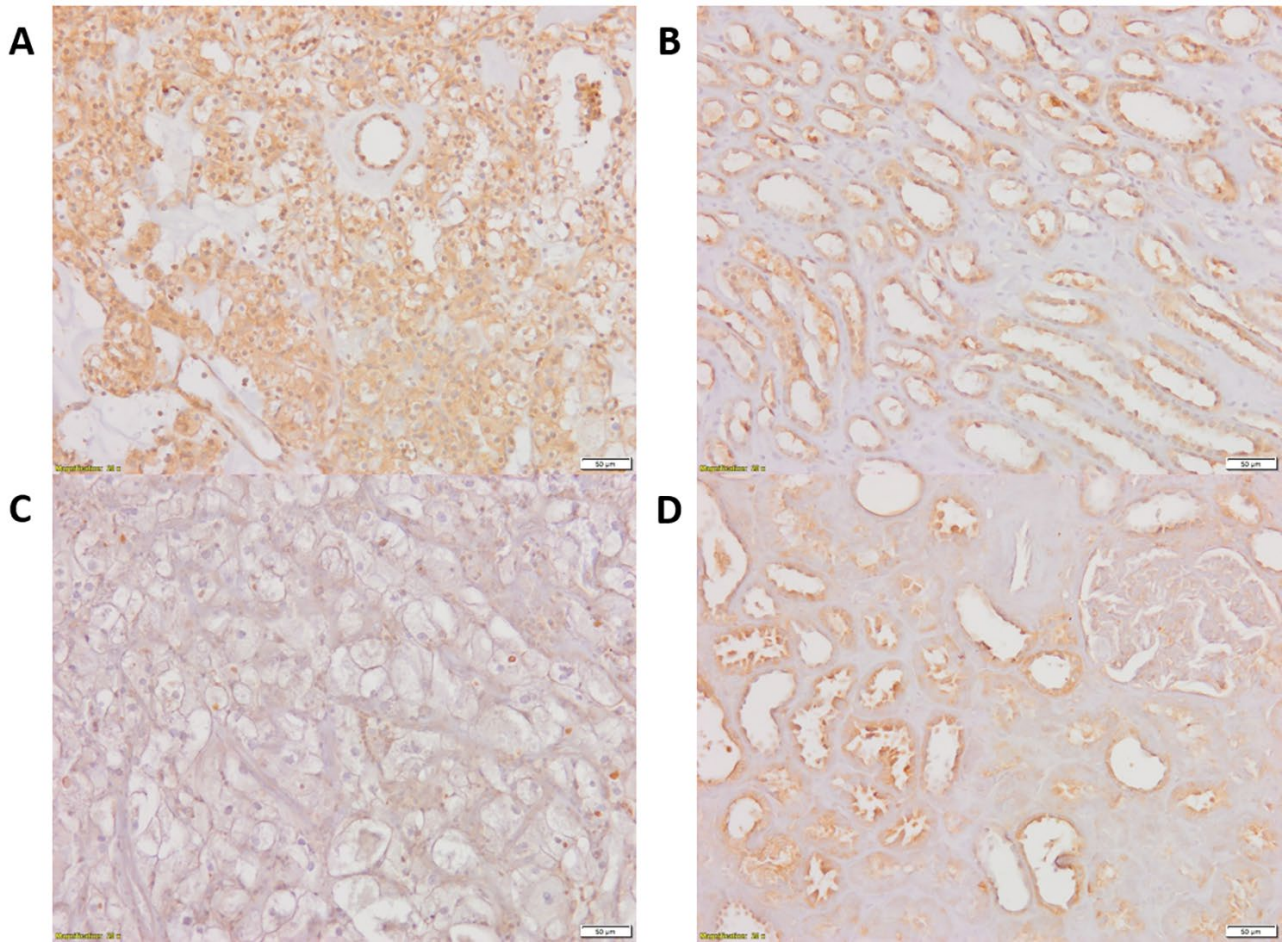
Representative microphotographs of GLI1 immunohistochemical reactions in KIRC samples (**A, C**) and corresponding morphologically unchanged kidney tissues (**B, D**). A – KIRC stage 1 (based on TNM classification), Fuhrman/WHO grade 3, follow-up period: 36 months; C - KIRC stage 3 (based on TNM classification), Fuhrman/WHO grade 3, death at 30 months of follow-up. Immunohistochemistry was carried out as described in Methods, sections were counterstained by hematoxylin. Scale bars, 50 μm. GLI1 - glioma-associated oncogene family zinc finger 1, KIRC - kidney renal clear cell carcinoma

**Fig. 2:**
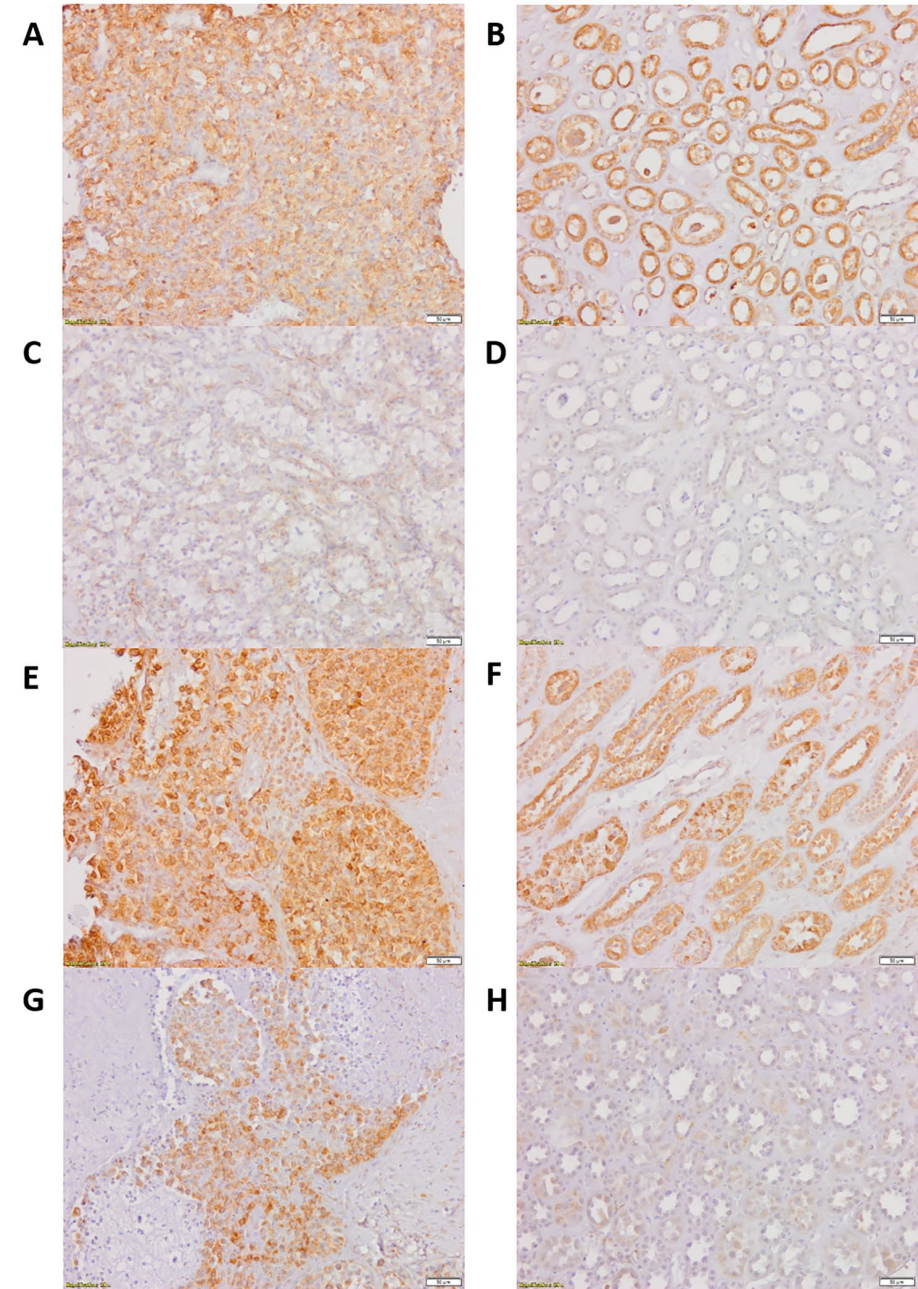
Representative microphotographs of SHH (**A, B, E, F**) and VEGFA (**C, D, G, H**) immureactivity in KIRC samples (**A, C, E, G**) and morphologically unchanged kidney (**B, D, F, H**). Slides **A, B, C, D** derived from the patient diagnosed with KIRC stage 1 (based on TNM classification), Fuhrman/WHO grade 3, follow-up period: 30 months; slides **E, F, G, H** derived from the patient diagnosed with KIRC stage 3 (based on TNM classification), Fuhrman/WHO grade 4, death at 6 months of follow-up. Immunohistochemistry was carried out as described in Methods, sections were counterstained by hematoxylin. Scale bars, 50 μm. SHH - sonic hedgehog, VEGFA - vascular endothelial growth factor A, KIRC - kidney renal clear cell carcinoma

Representative microphotographs that present comparison between immunoreactivity of GLI1 in paired tumor KIRC and unchanged kidney are shown in Fig. 1, while microphotographs demonstrating SHH and VEGFA immunoreactivity are shown in Fig. 2.

Cytoplasm was the main site of GLI1 location. Control and early (TNM 1 + 2) stages of KIRC revealed moderate and strong GLI1 IHC reaction, however, its intensity decreased in more advanced cancer samples (TNM 3 + 4). No association was found between tumor size, stage, grade, survival, and nuclear immunolocalization of GLI1 (data not shown). Control kidney samples demonstrated moderate and strong GLI1 IHC reaction in epithelial cells of the Bowman capsule, proximal and distal convoluted tubules, and collecting ducts.

Moderate and strong immunoreactivity of SHH was mainly observed in the cytoplasm and extracellular matrix (ECM) of cancer tissues. Control samples revealed strong SHH immunoreactivity in the epithelial cells of the Bowman capsule, proximal and distal convoluted tubules, collecting ducts, and thin limb of Henle’s loop.

VEGFA immunolocalization was associated with ECM and the cytoplasm of KIRC cells. The intensity of the VEGFA IHC reaction in cancer samples ranged from weak to strong. Control tissues demonstrate weak or moderate VEGFA immunoreactivity in epithelial cells of proximal convoluted tubules and collecting ducts as well as renal glomerulus cells.

**Fig. 3:**
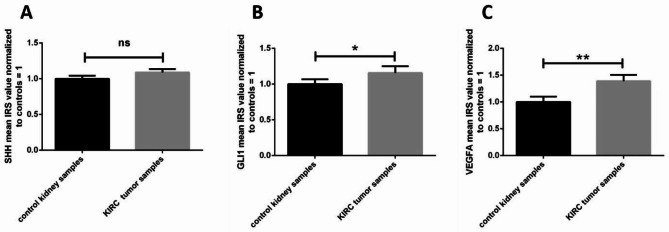
Analysis of the SHH, GLI1 and VEGFA immunoreactivity in KIRC. IRS was assessed as described in the Methods. Comparison between tumor and normal kidney samples. (**A**) – SHH protein, (**B**) – GLI1 protein, (**C**) – VEGFA protein. Bars and whiskers represent mean ± standard deviation normalized to control kidney samples. ^*^P < 0.05, **P < 0.01, Wilcoxon signed‑rank test. IRS – immunoreactivity score, SHH - sonic hedgehog, GLI1 - glioma-associated oncogene family zinc finger 1, VEGFA - vascular endothelial growth factor A, KIRC - kidney renal clear cell carcinoma

Direct comparison between KIRC tumor tissues and morphologically unchanged (control) kidney samples revealed a lack of statistical difference between the expression of SHH (Fig. 3A) and approximately 1.25- and 1.5 higher immunoreactivity of GLI1 and VEGFA proteins, respectively, in cancer cells (Fig. 3B,C). Further analysis of data in terms of patients’ clinical as well as KIRC pathological characteristics revealed a significant predominance of small tumors with elevated GLI1 levels, whereas there was no difference in GLI1 overall immunoexpression in large tumors (> 7 cm) (Table 1).

**Fig. 4:**
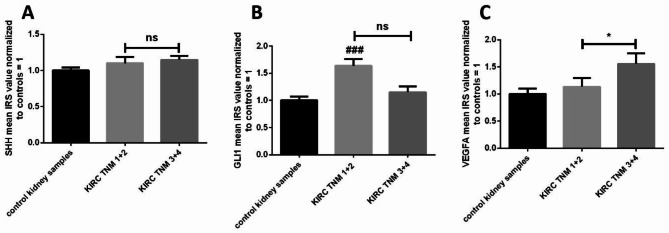
Immunoexpression analysis of the SHH, GLI1 and VEGFA proteins in KIRC classified by clinical TNM staging. IRS scoring was assessed as described in the Methods. Comparison between tumor and normal kidney samples; (**A**) – SHH protein, (**B**) – GLI1 protein, (**C**) – VEGFA protein. Bars and whiskers represent mean ± standard deviation normalized to control kidney samples. *P < 0.05, ### *P* < 0.001 between KIRC subgroup and control samples (Kruskal Wallis test). SHH - sonic hedgehog, GLI1 - glioma-associated oncogene family zinc finger 1, VEGFA - vascular endothelial growth factor A, KIRC - kidney renal clear cell carcinoma

**Fig. 5:**
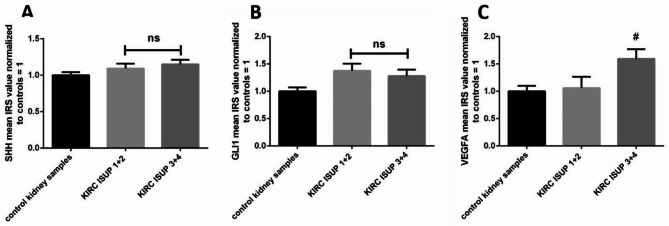
Analysis of the SHH, GLI1 and VEGFA immunoreactivity in KIRC samples classified by histological ISUP grading. IRS was assessed as described in the Methods. Comparison between tumor and normal kidney samples. (**A**) – SHH protein, (**B**) – GLI1 protein, (**C**) – VEGFA protein. Bars and whiskers represent mean ± standard deviation normalized to control kidney samples. # *P* < 0.05 between KIRC subgroup and control samples (Kruskal Wallis test). SHH - sonic hedgehog, GLI1 - glioma-associated oncogene family zinc finger 1, VEGFA - vascular endothelial growth factor A, KIRC - kidney renal clear cell carcinoma

The immunoreactivity of the analyzed proteins were not associated with patients’ age and sex (Table 1). No statistical association was found between SHH protein immunoexpression and tumor stage (Fig. 4A). However, the results obtained from KIRC samples and analyzed by Kruskal-Wallis test revealed the highest immunoreactivity of GLI1 protein in early clinically developed (TNM 1 + 2) KIRC samples, as compared to control tissue (Fig. 4B). Furthermore, we also observed significantly higher immunoreactivity of VEGFA in KIRC specimens assessed as TNM 3 + 4 compared to TNM 1 + 2 (Fig. 4C). The immunoexpression of SHH and GLI1 was not associated with tumor grade (Fig. 5A, B). However, we observed elevated immunoreactivity of VEGFA in ISUP 3 + 4 cancer tissues compared to control samples (Fig. 5C).

### The expression of SHH correlates with VEGFA expression in advanced KIRC

For correlation analysis of SHH, GLI1 and VEGFA immunoreactivity according to the tumor pathological characteristic and patients’ overall survival we divided patients into groups with clinically early/late, histologically benign/malignant and current state dead/alive (Table 2). The analysis revealed strong, positive (r > 0.5, *p* < 0.05) correlation between the expression of SHH ligand and VEGFA. However, this correlation was significant only for patients with advanced KIRC (TNM 3 + 4 and Fuhrman/WHO/ISUP 3 + 4) and samples derived from the patients who died during the follow-up period. No correlation was found between the immunoreactivity of GLI1 transcription factor and its potential target VEGFA as well as SHH, in KIRC tissues. Representative micrographs comparing SHH and VEGFA immunoexpression in early and advanced KIRC were shown in Fig. 2.

**Table 2:** Correlation between early/advanced KIRK stages, patient outcome and immunohistochemical pattern of SHH, GLI1 and VEGFA proteins’ expression in tumor tissues

Proteins	SHH		GLI1		VEGFA		SHH		GLI1		VEGFA	
Correlation results	r	*P*-value	r	*P*-value	r	*P*-value	r	*P*-value	r	*P*-value	r	*P*-value
**TNM 1 + 2**							**TNM 3 + 4**					
SHH	-		0.596	0.057	0.434	0.182	-		0.123	0.575	**0.571**	**0.004**
GLI1	0.596	0.057	-		0.315	0.341	0.123	0.575	-		-0.160	0.466
VEGFA	0.434	0.182	0.315	0.341	-		**0.571**	**0.004**	-0.160	0.466	-	
**WHO/Furhman 1 + 2**							**WHO/Furhman 3 + 4**					
SHH	-		-0.168	0.588	0.337	0.307	-		0.360	0.091	**0.532**	**0.009**
GLI1	-0.168	0.588	-		-0.137	0.669	0.360	0.091	-		-0.059	0.790
VEGFA	0.337	0.307	-0.137	0.669	-		**0.532**	**0.009**	-0.059	0.790	-	
**Survival - yes**							**Survival - no**					
SHH	-		0.397	0.103	0.411	0.090	-		0.245	0.261	**0.564**	**0.005**
GLI1	0.397	0.103	-		0.160	0.527	0.245	0.261	-		-0.077	0.728
VEGFA	0.411	0.090	0.160	0.527	-		**0.564**	**0.005**	-0.077	0.728	-	

The immunoexpression of proteins was analyzed by calculation of immunoreactivity scores (IRS) as described in Methods. r and P‑values were calculated by Spearman’s test: results with statistically significant values are indicated in bold print

### Low GLI1 and high VEGFA immunoreactivity in KIRC tissues are associated with shorter OS

**Fig. 6:**
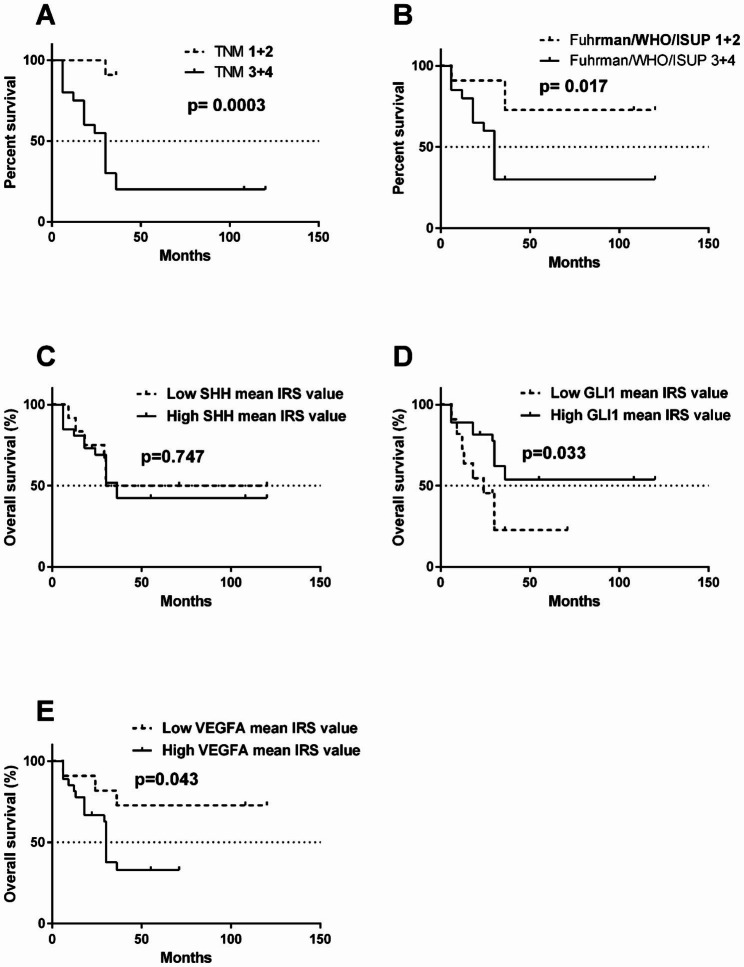
Kaplan-Meier’s overall survival analysis for KIRC patients in relations to clinical data and immunoexpression of SHH, GLII and VEGFA proteins. (**A**) TNM classification. (**B**) ISUP grade. (**C**) SHH, (**D**) GLI1, (**E**) VEGFA protein immunoexpression, respectively. Cut-off values between increased and decreased immunoreactivity scores were arbitrarily classified using median expression values of each protein in control samples. Log-rank (Mantel-Cox) test was applied

It was found that tumors characterized by advanced TNM stages and ISUP grades were associated with shorter OS (Fig. 6A, B) with a 50% survival rate of 30 months. No statistical association was found between SHH protein immunoexpression and patients’ OS (Fig. 6C). Shorter OS was significantly associated with lower GLI1 immunoreactivity (Fig. 6D) as well as high VEGFA immunoexpression (Fig. 6E).

### Advanced cancer and GLI1 as well as VEGFA expression patterns are risk factors in KIRC

Cox proportional hazards test with univariable and multivariable regression analyses revealed that patients with advanced TNM stages, high ISUP grade, low GLI1 and high VEGFA immunoreactivity were associated with high risk of death in the course of KIRC, as shown in Table 3.

**Table 3:** Univariable and multivariable Mantel-Cox regression analysis of the overall survival rate of kidney renal cancer patients

Parameters	Univariable analysis			Multivariable analysis		
	χ2	*P*-value	HR (95 CI)	χ2	*P*-value	HR (95 CI)
Sex Female vs. Male	0.188	0.664	1.186 (0.550–2.557)			
Age (years) > 67 vs. ≤67	0.416	0.519	0.728 (0.277–1.913)			
Tumor size (cm) > 7 vs. ≤7	2.259	0.133	0.565 (0.268–1.190)			
Tumor stage T3 + 4 vs. T1 + 2	**8.573**	**0.0034**	**3.157** **(1.462–6.817)**	**4.528**	**0.033**	**2.352 (1.069–5.171)**
Histological ISUP grade 3 + 4 vs. 1 + 2	**13.093**	**0.0003**	**5.082** **(2.106–12.262)**	**9.694**	**0.0018**	**4.156 (1.695–10.192)**
SHH IRS value ↑ vs. ↓	0.408	0.523	0.732 (0.281–1.907)			
GLI1 IRS value ↓ vs. ↑	**4.001**	**0.045**	**2.488 ** **(1.004–6.169)**	**3.884**	**0.048**	**2.798 (1.005–7.785)**
VEGFA IRS value ↑ vs. ↓	**4.199**	**0.0404**	**3.725 ** **(1.286–13.108)**	**3.644**	**0.049**	**3.3716 (0.951–11.951)**

Downward and upward arrows indicate decreased and increased levels of SHH, GLI and VEGF immunoreactivity. Values in bold indicate statistical significance (*P* < 0.05)

## Discussion

Although new therapies such as tyrosine kinase inhibitors (TKI) target therapy or immunotherapy have been implemented for KIRC treatment, this disease continues to show global high mortality rates [1]. The involvement of the VHL-HIF-VEGF axis in kidney cancer development was detected in the 1990s [42, 43]. This discovery has led to the introduction of drugs such as sunitinib and sorafenib into KIRC therapy which significantly increased patient survival periods [44–46]. The association of SHH pathway activity and *VEGFA* expression [24] prompted us to consider the involvement of these molecular factors in the pathogenesis of KIRC, particularly in terms of their prognostic significance. Our previous research focused on the expression of SHH pathway components and their targets at the mRNA level and we observed their upregulation in early KIRC samples [35]. Therefore, we decided to investigate the immunoreactivity of the two major SHH pathway proteins, SHH and GLI1, as well as the expression of one of their important targets - VEGFA, in this type of renal cancer.

The immunoreactivity of GLI1 transcription factor was increased only in patients at early KIRC stage (TNM stages 1 + 2). This result is in line with our previous data obtained for *GLI1* expression at the mRNA level that was increased in 25/33 KIRC patients at the early stage of disease [35]. To the best of our knowledge this is the first report concerning elevated immunoreactivity of GLI1 protein in early stages of KIRC. Furthermore, low GLI1 immunoreactivity correlated with shorter patients’ overall survival, suggesting its prognostic potential in KIRC. The study of Zhou et al. presented the data regarding the immunoreactivity of GLI transcription factors in renal cell carcinoma [47]. According to the comparison of GLI1 level in cancer and control samples, higher expression was associated with KIRC samples what is consistent with our results. However high GLI1 immunoreactivity was related to shorter overall survival period of patients [47]. Association between low nuclear GLI1 immunoexpression and shorter progression-free survival was observed in human glioblastoma samples [48]. It was also found that low-grade urinary bladder tumors were more likely to stain for GLI1 as compared with high-grade tumors [49]. Moreover, non–muscle-invasive bladder tumors expressing GLI1 were less likely to recur than those in which GLI1 was absent [49]. These reports point to a possible oncogenic role for the GLI1 protein. Some of the mentioned results are contrary to ours, since we clearly found a possible GLI1 protective role in KIRC, based on the Cox test where samples with downregulated GLI1 immunoreactivity were associated with earlier death in the course of this cancer type. Nonetheless, given the discrepancies presented in the studies, more research is needed to establish the role of GLI1 in KIRC as well as other types of cancer.

VEGFA immunoreactivity was increased in KIRC tissue, especially in samples assessed as ISUP 3 + 4 compared to morphologically unchanged kidney tissue. Moreover, we observed the association between elevated level of VEGFA and patients’ shorter overall survival. Those results support our previous findings regarding the expression of VEGFA at the mRNA [35] and protein levels [3]. However, no correlation was found between GLI1 and VEGFA immunoreactivity neither in early (TNM 1 + 2, WHO/Fuhrman 1 + 2) nor advanced KIRC samples (TNM 3 + 4, WHO/Fuhrman 3 + 4). Potential causes of this findings may be due to stronger influence of HIFs or other molecular factors on the *VEGFA* gene stimulation than SHH signaling in KIRC [32, 50]. Moreover, disturbances in the functioning of GLIs as transcription factors may be caused by their incorrect activation as a result of morphological abnormalities of the primary cilium in cancer cells [51]. Increased *VEGFA* expression at the mRNA level in KIRC tumor samples compared to normal kidney tissues was also found in tissues from a large group of patients (research based on mRNA-sequencing data of KIRC from an online database) [52]. Moreover, Crona et al. identified potential *VEGFA* genetic variants that could be responsible for shorter OS in renal cell carcinoma patients treated with sorafenib [53]. Thus, VEGFA can be considered as a potential prognostic factor whose higher expression is associated with a worse prognosis for KIRC patients, as showed by both Kaplan‑Meier and Cox tests.

One of the novel findings in our study is the positive correlation between SHH and VEGFA levels in advanced KIRC (TNM 3 + 4, WHO/Fuhrman 3 + 4). We suggest that a non-canonical, GLI-independent pathway of SHH protein activity [54] may be responsible for this relationship. Similarly to us, Huljev et al. demonstrated the trend of a linear decrease of the SHH immunofluorescence with the progression of the KIRC tumour grade [55]. Reports regarding SHH protein immunoreactivity in other types of cancer are inconsistent. For instance, in a Japanese study on gastric cancer high SHH protein immunoreactivity assessed by IHC was associated with poor prognosis [56]. However, on the contrary, a Korean study involving larger cohort of gastric cancer patients, revealed longer overall survival in a group with overexpression of SHH demonstrated by IHC staining [57]. The reason for these discrepancies may result from post-translational modifications of the SHH protein, as our previous analysis of SHH protein levels by western blot technique in KIRC tissues, showed differences between levels of the full-length SHH molecule and C-terminal SHH domain [58]. Thus, subsequent studies of SHH expression should take into account the post-translational SHH processing.

A limitation of our study is a relatively small number of participants. However, it has to be noted that the clinical features such as mean age and M/F ratio correspond with RCC global epidemiology [33].

## Conclusions

In summary, the results of our analyzes are the first to reveal that the decrease in the immunoexpression of the transcription factor GLI1 in KIRC is associated with shorter patient survival confirming our previous findings at the mRNA level. Immunohistochemical evaluation of GLI1 and VEGFA reactivity should be considered as a prognostic marker in KIRC.

## Data Availability

The datasets used and/or analyzed during the current study are available from the corresponding author on reasonable request.

## List of abbreviations

CI: confidence interval
ECM: extracellular matrix
GLI1: glioma-associated oncogene family zinc finger 1
GLI2/GLI3-SUFU-KIF7: glioma-associated oncogene family zinc finger 2/3- suppressor of fused- kinesin family member 7
HIF: hypoxia inducible factor
HRs: hazard ratios
IHC: immunohistochemical
IRS: immunoreactive score
KIRC: kidney renal clear cell carcinoma
OS: overall survival
PBS: phosphate-buffered saline
PDGF: platelet-derived growth factor
PTCH1: patched 1
RCC: renal cell carcinoma
SHH: sonic hedgehog
SMO: smoothened
TFs: transcription factors
TKI: tyrosine kinase inhibitors
VEGFA: vascular endothelial growth factor A
VHL: von Hippel–Lindau
VHL-HIF-VEGF: von Hippel–Lindau-hypoxia inducible factor-vascular endothelial growth factor pathway
